# Inspiratory Strength and Diaphragm Thickness in Tennis Players with and Without Non-Specific Shoulder Pain

**DOI:** 10.3390/jcm15176541

**Published:** 2026-08-24

**Authors:** Davinia Vicente-Campos, Jorge Sánchez-Infante, César Calvo-Lobo, David Rodríguez-Sanz, Ricardo Becerro-de-Bengoa-Vallejo, José López-Chicharro, Iñaki Quintana

**Affiliations:** 1 Institute of Health and Sport Sciences, Faculty of Health Sciences, Universidad Francisco de Vitoria, 28223 Pozuelo de Alarcón, Spain; davinia.vicente@ufv.es; 2 Physiotherapy Research Group of Toledo (GIFTO), Faculty of Physiotherapy and Nursing, Universidad de Castilla-La Mancha, 45071 Toledo, Spain; 3 Faculty of Nursing, Physiotherapy and Podiatry, Universidad Complutense de Madrid, 28040 Madrid, Spain; cescalvo@ucm.es (C.C.-L.); davidrodriguezsanz@ucm.es (D.R.-S.); ribebeva@enf.ucm.es (R.B.-d.-B.-V.); joselg19@ucm.es (J.L.-C.); 4 Grupo de Investigación TECNODEF, Faculty of Health Sciences, Universidad Internacional de La Rioja (UNIR), 26006 Logroño, Spain; inaki.quintana@unir.net

**Keywords:** athletic injuries, sports medicine, ultrasonography, physical therapy modalities, respiratory muscles

## Abstract

**Background/Objectives:** Shoulder pain in tennis players is usually interpreted from a local shoulder perspective, although kinetic-chain and trunk-control factors may also be relevant. The diaphragm contributes to breathing and trunk stabilization, but diaphragm-related measures have received limited attention in tennis players with shoulder pain. This study compared inspiratory muscle strength, diaphragm thickness, and diaphragm thickening fraction between tennis players with and without non-specific shoulder pain. **Methods:** This observational cross-sectional study included 64 tennis players: 32 with non-specific shoulder pain and 32 asymptomatic controls. Maximum inspiratory pressure (MIP), bilateral diaphragm thickness during relaxed inspiration and expiration, and diaphragm thickening fraction were assessed. Between-group differences were analyzed. **Results:** Tennis players with non-specific shoulder pain showed lower MIP than controls (*p* = 0.002). Diaphragm thickness during relaxed inspiration was lower in symptomatic players on the left (false discovery rate (FDR)-adjusted *p* = 0.033) and right sides (FDR-adjusted *p* = 0.012), and diaphragm thickening fraction was also lower bilaterally (left: FDR-adjusted *p* = 0.036; right: FDR-adjusted *p* = 0.039). **Conclusions:** Tennis players with non-specific shoulder pain showed lower inspiratory muscle strength, lower bilateral diaphragm thickness during relaxed inspiration, and lower bilateral diaphragm thickening fraction than asymptomatic players. These cross-sectional findings describe group-level differences but do not establish causality, diaphragm dysfunction, or clinical relevance.

## 1. Introduction

Shoulder pain is a frequent musculoskeletal complaint worldwide and is considered one of the most prevalent pain conditions, together with low back and knee pain [1]. Epidemiological studies have reported that, in the general population, shoulder pain may persist for 6–12 months in up to 50% of patients, and rotator cuff-related shoulder pain accounts for approximately 50–85% of shoulder pain diagnoses [2]. Several risk factors may compromise shoulder function and contribute to the development or persistence of shoulder pain including previous history of shoulder pain, with or without an associated structural injury, reduced shoulder range of motion and flexibility, overuse, age, humeral and scapular asymmetries, increased joint laxity, scapular dyskinesis, and muscle weakness or imbalance [3,4,5]. In addition, the mechanisms underlying shoulder pain may vary according to individual occupational demands, training load, sport-specific gestures, and other activity-related factors [6,7,8]. For instance, in racket sports, shoulder pain is highly prevalent (with reported rates of 15.9% in tennis and 8.4% in padel [2,9,10]).

Despite its high prevalence, shoulder pain is frequently described as “non-specific”, a term that reflects the difficulty of identifying a precise anatomical or pathophysiological source of symptoms. Although shoulder pain is often suspected to be related to common clinical entities such as rotator cuff-related disorders, which may account for up to 65% of cases and have an estimated prevalence of approximately 20%, tendinopathy, or subacromial impingement, determining the exact cause of pain remains challenging [7]. In this line, previous articles found that many racket sport players report shoulder pain despite the absence of a clearly defined structural injury [11,12] (approximately, 38.5% are musculotendinous injuries, of which 7.8% involve the shoulder rotator cuff complex). Although these injuries are generally mild and intrinsic in origin, and approximately 40% do not require medical or physiotherapy care, they may still result in pain that limits performance; conversely, they may also remain minimally symptomatic, allowing players to continue playing without major functional limitations [11].

Tennis also involves specific contextual and biomechanical demands that may increase shoulder injury risk. Tennis is an intermittent racket sport characterized by repeated multidirectional movements and unilateral high-velocity strokes, including serves and overhead actions that require coordinated force transfer through the kinetic chain [3,13]. Some of these factors are external to the athlete, including the unpredictable duration of matches, long competitive seasons, high competitive demands, and insufficient recovery time between tournaments [4,5,8]. Others are related to sport-specific factors, such as equipment selection, since the characteristics of the racket or padel racket may influence mechanical load and therefore contribute to injury prevention [11,12]. Furthermore, shoulder injuries appear to occur more frequently in players with suboptimal technique, inadequate physical conditioning, or insufficient strength preparation, which may increase the susceptibility of the upper limb, particularly the elbow and shoulder joints, to injury [5,10,11]. In tennis players, imbalance between the internal and external rotators may be particularly relevant, as the internal rotators are typically stronger than the external rotators; therefore, specific preventive strategies targeting shoulder muscle balance should be incorporated into training programs to reduce injury risk [14].

Beyond local shoulder impairments, shoulder pain in tennis players should also be interpreted within the broader context of kinetic chain function. Tennis strokes require the coordinated activation of the lower limbs, trunk, and upper limb, with force being progressively transferred through the core towards the shoulder and racket. If energy transfer from the trunk or lower limbs is insufficient, the upper limb may need to compensate for this deficit, potentially increasing mechanical stress on the shoulder and compromising stroke velocity, power, and movement efficiency [5,13,15]. Therefore, alterations in trunk control or core function may be relevant contributors to shoulder overload in tennis players. In this regard, previous evidence has linked altered diaphragm function not only with shoulder-related symptoms but also with low back pain, supporting its potential role in musculoskeletal conditions involving trunk control and kinetic chain function [16]. The diaphragm may be particularly important in this context because it has a dual role as both the primary inspiratory muscle and a key contributor to trunk stabilization. Beyond its inspiratory role during quiet breathing, the diaphragm interacts with deep stabilizing muscles of the trunk to contribute to lumbopelvic stability, postural control, and force transmission across adjacent regions [17]. This stabilizing role is especially relevant during rapid voluntary limb movements, where anticipatory activation of the core musculature helps prepare the trunk for movement and supports efficient force transmission along the kinetic chain [18].

Based on this rationale, this study compared inspiratory muscle strength, diaphragm thickness, and diaphragm thickening fraction between tennis players with and without non-specific shoulder pain.

## 2. Materials and Methods

### 2.1. Study Design

This observational cross-sectional comparative study was carried out from November 2025 to February 2026. Recruitment and assessment procedures took place at the Francisco de Vitoria University Campus, within the Faculty of Health Sciences, and at the Madrid Tennis Federation. Group classification and outcome measurements were obtained during the same assessment period, and no longitudinal follow-up was performed. To enhance reporting quality and methodological transparency, the manuscript was prepared in accordance with the STROBE guidance for observational studies [19].

### 2.2. Participants

The study sample comprised two groups of tennis players: asymptomatic players and players with non-specific shoulder pain. Before participation, all subjects received detailed information about the study. Participants in both groups were eligible if they were aged 18–60 years and played tennis at least twice per week. Participants were excluded if they reported (1) any shoulder pain associated with dislocation, subluxations, fractures, or radiculopathies; (2) history of shoulder or thoracic surgeries; (3) any previously diagnosed medical condition explaining their shoulder pain; (4) any diagnosed respiratory or neurological pathology; (5) any heart failure; (6) any psychological condition; or (7) any difficulty or inability to understand and sign the informed consent form.

Specific inclusion criteria for the shoulder pain group were (1) non-specific shoulder pain while playing and in the month prior to the start of data collection and (2) pain scores equal to or greater than 3 on the Visual Analogue Scale (VAS, which is considered the minimal clinically relevant threshold [20]). For the purposes of this study, “non-specific shoulder pain” was operationally defined as shoulder pain during tennis, present during the month before data collection, with a VAS score ≥ 3 and without a previously diagnosed condition explaining the symptoms. The term was not intended to represent a specific clinical diagnosis or to imply that all identifiable shoulder conditions had been ruled out. No standardized shoulder examination or specific clinical test battery was performed as part of the study.

An a priori sample-size calculation was performed with G*Power version 3.1.9.2 (Heinrich Heine University Düsseldorf, Düsseldorf, Germany) for a two-tailed independent-samples comparison based on the primary outcome, maximum inspiratory pressure. As no previous study or pilot data were available to estimate the expected effect size in tennis players with shoulder pain, the conventional threshold proposed by Cohen for a large between-group effect (d = 0.80) was used. With α = 0.05, 80% power, and equal group allocation, the minimum required sample was 52 participants, with 26 tennis players with non-specific shoulder pain and 26 asymptomatic controls. This yielded an actual power of 0.807 [21]. The final sample included 64 participants, exceeding the minimum required sample size. As the study had a cross-sectional design, no follow-up losses were anticipated.

### 2.3. Data Collection

Assessments were conducted by three different researchers. The ultrasound examiner and the investigator assessing maximum inspiratory pressure (MIP) did not have access to participants’ VAS or QuickDASH scores or to their group classification at the time of assessment. A separate investigator administered the self-reported questionnaires and classified participants according to the predefined eligibility criteria. Participant identities and data were coded using alphanumeric identifiers before statistical analysis. The ultrasound examiner had more than five years of experience in musculoskeletal ultrasound imaging (US) and acquired and quantified all diaphragm images.

MIP was considered the primary outcome because it provides a global measure of inspiratory muscle strength. Its between-group comparison was considered the primary analysis. Bilateral diaphragm thickness during relaxed inspiration and expiration and diaphragm thickening fraction were considered secondary exploratory outcomes. The VAS, QuickDASH, and QuickDASH Sports questionnaires were administered only to participants in the shoulder pain group to characterize pain intensity and shoulder-related disability. These questionnaires were not completed by the asymptomatic control group.

#### 2.3.1. Pain Intensity: Visual Analogue Scale

The Visual Analogue Scale (VAS) was used to quantify pain intensity. Participants placed a mark on a 10-cm horizontal scale, with the left endpoint representing no pain and the right endpoint representing the worst imaginable pain. Scores were calculated as the distance, in centimetres, from the no-pain endpoint to the marked point [22].

#### 2.3.2. Shoulder Disability and Function: QuickDASH

Shoulder-related disability was evaluated with the Shortened Disabilities of the Arm, Shoulder and Hand Questionnaire (QuickDASH) [23]. This self-administered instrument includes 11 items addressing symptoms and functional limitations of the upper limb. Responses are rated on a 5-point scale and converted to a 0–100 score, where higher values reflect greater disability. A valid score requires completion of at least 10 items. The QuickDASH was derived from the original 30-item DASH to reduce respondent burden while maintaining comparable measurement properties [24]. Previous studies have supported its reliability, validity, and responsiveness in individuals with upper-limb musculoskeletal disorders [25].

In addition, the optional QuickDASH Sports Module was administered to assess sport-specific upper-limb disability. This module is designed for individuals whose symptoms may affect higher-demand activities such as sport or performing arts, and it provides complementary information to the core QuickDASH score by focusing on functional limitations during sport-related tasks. As with the main QuickDASH, items are scored on a 5-point Likert scale and transformed into a 0–100 score, with higher values indicating greater disability. Its inclusion was considered appropriate for the present sample because athletes often present high baseline upper-extremity function, and sport-specific modules may help capture functional limitations that are not fully reflected by general activities of daily living [26].

#### 2.3.3. Diaphragm Thickness: Ultrasound Imaging

Diaphragm thickness was assessed by B-mode ultrasound using a Canon Aplio a device equipped with a linear PLT-1005-BT 14L5 transducer (5–14 MHz) (Canon Medical Corp, Otawara, Tochigi, Japan). The same ultrasound configuration was used for all participants, including a depth of 4.5 cm, frequency of 11 MHz, dynamic range of 70, gain of 78, and a single focal point placed at 2 cm depth.

Participants were examined in the supine position. The transducer was placed perpendicular to the lower intercostal space on the midaxillary line, between the superior border of the 12th rib and the inferior border of the 11th rib. This location was selected to optimize visualization of the diaphragm at the zone of apposition during tidal breathing while minimizing lung interference [27,28].

**Figure 1:**
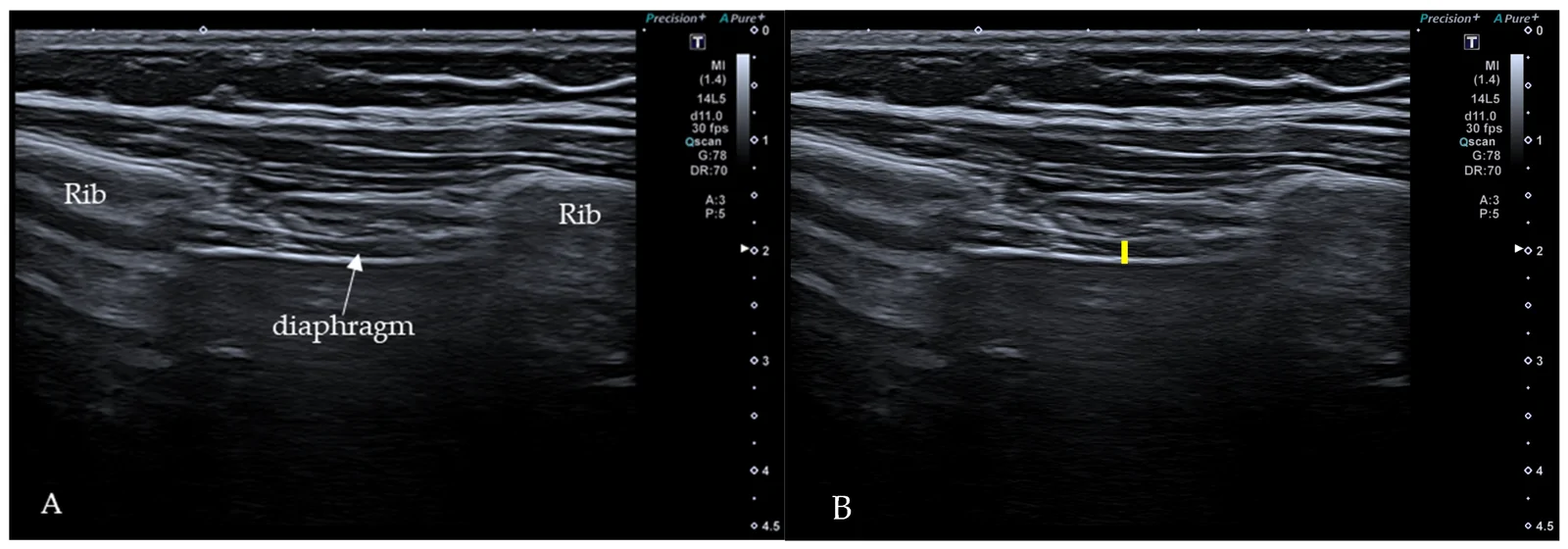
(**A**) Representative B-mode ultrasound image of the diaphragm obtained with a linear transducer over the lower intercostal space on the midaxillary line. (**B**) Measurement of diaphragm thickness at the zone of apposition. The diaphragm appears as a hypoechoic layer between the pleural and peritoneal hyperechoic lines. Yellow calipers indicate the distance used to quantify diaphragm thickness.

Bilateral diaphragm thickness was quantified according to previously described procedures with excellent inter-rater reliability, assessed using the intraclass correlation coefficient (ICC = 0.94–0.98) [29]. The diaphragm was visualized as a hypoechoic muscle layer situated beneath the intercostal muscles and delimited by two hyperechoic borders corresponding to the pleural and peritoneal membranes (Figure 1). Thickness was measured with electronic calipers positioned between these two borders at the midpoint of the intercostal space. Measurements were obtained for each hemidiaphragm at the visually identified end of relaxed expiration (Texp) and inspiration (Tins) during quiet tidal breathing, without forced or maximal respiratory maneuvers. Tidal volume was not simultaneously monitored or instrumentally standardized. Three images were recorded for each side and respiratory phase, and the average of the three measurements was used for statistical analysis [27]. Diaphragm thickening fraction (DTF) was calculated separately for each hemidiaphragm from the averaged thickness values using the following formula: DTF (%) = [(Tins − Texp)/Texp] × 100.

#### 2.3.4. Inspiratory Muscle Strength: Maximum Inspiratory Pressure

Inspiratory muscle strength was assessed as MIP using a POWERbreathe^®^ KH1 device (POWERbreathe International Ltd., Southam, Warwickshire, UK), following international recommendations for respiratory muscle testing [30,31]. The device was used within the manufacturer-recommended calibration period. Participants were seated with their hips and knees flexed at 90°. They were instructed to perform a slow and prolonged expiration down to residual lung volume, followed by a maximal forced inspiration through the device while wearing a nasal clip and keeping the glottis open [30]. Participants were also instructed not to place or bite the tongue against the mouthpiece and to maintain an airtight seal with their lips to prevent air leakage during the maneuver.

Several days before the assessment, participants completed a familiarization session to ensure that they understood and could correctly perform the maneuver. On the assessment day, the same standardized instructions and verbal encouragement were provided to all participants. Two measurements were performed with a one-minute rest interval, and the highest value was retained for analysis when the difference between attempts did not exceed 10%. When this threshold was exceeded, a third measurement was performed, and the highest value was retained [31]. MIP was reported as an absolute value because no sport-specific reference equations validated in tennis players are currently available. Accordingly, these values were used for between-group comparison and not to classify participants as having clinical inspiratory muscle weakness.

#### 2.3.5. Statistical Analysis

All analyses were carried out in SPSS v.31 (IBM Corp., Armonk, NY, USA). Statistical significance was established at *p* < 0.05, and results were reported with 95% confidence intervals (CI). According to Lilliefors correction, the Kolmogorov–Smirnov test was used to determine normal distribution. All data were described as mean ± standard deviation (SD) completed with 95% CI (lower and upper limits). Differences for parametric data between non-specific shoulder pain tennis players and asymptomatic tennis players were analyzed using the Student’s *t*-test for independent samples following the Levene’s test for equality of variances. For variables that did not meet normality assumptions, between-group differences were examined using the Mann–Whitney U test. Means and standard deviations were retained for all continuous outcomes to ensure consistent descriptive reporting across variables. For outcomes analyzed with the Mann–Whitney U test, statistical inference was based on ranks, whereas mean differences and Cohen’s d were reported as complementary descriptive measures of the magnitude of the between-group differences and not as test-specific effect estimates. Cohen’s d and its corresponding 95% confidence interval were calculated to estimate effect size magnitude. Effect sizes were classified as small (0.20–0.49), moderate (0.50–0.79), or large (≥0.80) [32]. Maximum inspiratory pressure was treated as the primary outcome and was tested at a two-sided significance level of 0.05. The six diaphragm ultrasound comparisons were treated as secondary exploratory analyses. To account for multiple testing across the six secondary diaphragm ultrasound outcomes, *p*-values were adjusted using the Benjamini–Hochberg false discovery rate procedure. Both unadjusted and FDR-adjusted *p*-values are reported. ChatGPT (GPT-5, OpenAI) was used solely for English-language editing during manuscript preparation.

## 3. Results

Descriptive and clinical characteristics, including estimated between-group differences, 95% confidence intervals, and effect sizes, are presented in Table 1, while comparisons of the main outcome measures are shown in Table 2. No formal equivalence testing was performed, and non-significant *p*-values were not interpreted as evidence of baseline homogeneity. The shoulder pain group included 26 men and 6 women, whereas the asymptomatic group included 25 men and 7 women. All participants were right-hand dominant and used the right arm as their racket-playing side. All participants in the shoulder pain group reported pain in the dominant right shoulder.

Regarding the outcome measures, tennis players with non-specific shoulder pain showed significantly lower MIP compared with asymptomatic tennis players (*p* = 0.002), indicating reduced inspiratory muscle strength. In addition, diaphragm thickness during relaxed inspiration was significantly lower bilaterally in the shoulder pain group, both on the left side (*p* = 0.011) and on the right side (*p* = 0.002). Diaphragm thickening fraction was also significantly lower bilaterally in the shoulder pain group (left: *p* = 0.018; right: *p* = 0.026). The magnitude of these differences was moderate to large, indicating that the observed between-group differences were not trivial. All significant differences in the secondary diaphragm ultrasound outcomes remained statistically significant after adjustment using the Benjamini–Hochberg false discovery rate procedure (Table 2).

In contrast, diaphragm thickness during relaxed expiration did not differ significantly between groups on either side (*p* > 0.05), with small effect sizes. Therefore, the main between-group differences were observed in inspiratory-related measures rather than in expiratory diaphragm thickness. Overall, these findings indicate that tennis players with non-specific shoulder pain showed lower inspiratory muscle strength, inspiratory diaphragm thickness, and diaphragm thickening fraction than asymptomatic tennis players.

## 4. Discussion

The findings of this study suggest that tennis players with non-specific shoulder pain present lower inspiratory muscle strength compared with asymptomatic tennis players. Symptomatic players showed lower MIP, lower bilateral diaphragm thickness during relaxed inspiration, and lower bilateral diaphragm thickening fraction, whereas diaphragm thickness during relaxed expiration did not significantly differ between groups. These findings represent group-level differences and do not establish a temporal or mechanistic relationship between shoulder pain and diaphragm-related measures.

These findings may be relevant to the broader assessment of shoulder pain in tennis players, which cannot always be fully understood from a local anatomical perspective alone. Although shoulder symptoms are often interpreted in relation to rotator cuff disorders, tendinopathy, impingement-related mechanisms, or local muscle imbalance, the relationship between structural findings and pain is frequently inconsistent [33,34]. Previous literature has also highlighted that shoulder pain and functional limitation do not always correlate closely with structural findings, and that asymptomatic individuals may present structural shoulder abnormalities [15]. Therefore, broader functional interactions involving the trunk, scapula, respiratory system, and kinetic chain may be relevant in athletes with non-specific shoulder pain.

In this context, the muscle chain and kinetic-chain models provide a useful framework to interpret the present results. Human movement depends on the coordinated interaction of joint chains, muscle chains, and neural chains, rather than on the isolated action of individual muscles [15]. Muscle chains, including synergistic muscle groups, muscle slings, and myofascial chains, contribute to force transmission, postural control, and movement efficiency [35]. This is particularly relevant in tennis, where the serve and overhead strokes require the sequential transfer of force from the lower limbs and pelvis through the trunk and scapulothoracic region towards the shoulder, arm, and racket [3].

The diaphragm may be an important component of this system because it has a dual function as the main inspiratory muscle and as a contributor to trunk stabilization, together with the deep abdominal wall, pelvic floor, and deep posterior trunk muscles [36]. Lv, S., et al. [15] emphasized that breathing movement is closely related to postural control and limb movement, and that changes in shoulder girdle position, cervical–thoracic mobility, scapular mechanics, and accessory respiratory muscle activity may influence breathing mechanics. In tennis players, repeated overhead actions, trunk rotation, and high-velocity force transfer require coordinated interaction between respiratory and postural functions [13]. Therefore, the observed differences in inspiratory measures may reflect a broader interaction between shoulder pain, scapulothoracic adaptations, trunk control strategies, and respiratory muscle behavior. However, this interpretation remains hypothetical because these mechanisms were not directly assessed.

The absence of significant between-group differences in expiratory diaphragm thickness, together with the lower inspiratory thickness and thickening fraction observed bilaterally, suggests that the main between-group differences emerged during the inspiratory phase rather than in baseline expiratory thickness. The lower thickening fraction indicates reduced relative diaphragm thickening from expiration to inspiration in tennis players with shoulder pain [15,17]. However, these findings should not be interpreted as direct evidence of impaired diaphragm contractility, as thickening measurements may be influenced by breathing pattern, tidal volume, and lung volume during image acquisition.

From a biomechanical perspective, shoulder pain may be accompanied by protective movement strategies involving the scapulothoracic and cervical regions. These adaptations could hypothetically influence breathing mechanics and inspiratory muscle behavior [15]. However, this explanation remains speculative because accessory respiratory muscle activation, thoracic mobility, and scapular kinematics were not directly assessed.

Clinical evidence also supports the potential relevance of the diaphragm in shoulder pain. In a randomized controlled trial including participants with rotator cuff injury, manual therapy directed at the diaphragm produced clinically significant improvements in shoulder pain during flexion and abduction, as well as improvements in pressure pain threshold at the xiphoid process [37]. Although these studies were not conducted in tennis players and do not establish causality, they support the idea that the diaphragm may be clinically relevant in some shoulder pain presentations.

Differences in diaphragm-related measures have also been reported in other musculoskeletal pain populations, including patients with chronic painful temporomandibular disorders [38]. Although this provides contextual support for studying respiratory–postural interactions, these findings cannot be directly extrapolated to tennis players with shoulder pain.

Shoulder pain in tennis players is multifactorial and may be influenced by training load, previous injury, stroke technique, scapular control, rotator cuff strength, thoracic mobility, psychological factors, recovery status, and other individual characteristics [4,5,10,11,13]. Therefore, diaphragm-related measures should not be interpreted as diagnostic markers or isolated treatment targets.

From a clinical perspective, the present findings do not support routine diaphragm-focused assessment or treatment in tennis players with shoulder pain because diagnostic utility, prognosis, and intervention effectiveness were not evaluated. Moreover, established minimal clinically important differences and minimal detectable changes for diaphragm thickness and thickening fraction in tennis players are not available; therefore, the statistically significant between-group differences observed in this study cannot be directly interpreted as clinically meaningful. Previous conceptual work has proposed that respiratory and postural factors may be considered within broader shoulder rehabilitation frameworks [15]. However, the present findings do not establish these factors as clinical targets. Inspiratory muscle strength and diaphragm-related ultrasound measures should therefore be considered variables for further investigation rather than established components of clinical assessment or treatment. Longitudinal and interventional studies are required before specific recommendations regarding respiratory or diaphragm-focused assessment and rehabilitation can be made.

Several limitations should be acknowledged. First, the cross-sectional design prevents causal interpretation, and it cannot be determined whether the observed inspiratory differences preceded shoulder pain or resulted from pain-related adaptations. Second, diaphragm thickness and thickening fraction during relaxed breathing should not be interpreted as complete measures of diaphragm function. Tidal volume was not simultaneously monitored or instrumentally standardized, and the measurements may therefore have been influenced by breathing pattern and lung volume during image acquisition. In addition, intra-rater reliability was not specifically assessed in the present sample. Although participants completed a previous familiarization session, the absence of a formal plateau criterion during MIP assessment may not fully exclude effort-related or learning-related variability. Third, although participants with previously diagnosed conditions explaining their shoulder pain were excluded, no standardized clinical examination was performed; therefore, undiagnosed conditions and clinical heterogeneity cannot be ruled out. Fourth, relevant demographic, respiratory, clinical, and tennis-specific confounders—including BMI, smoking status, pulmonary function, training exposure, competitive level, previous shoulder injury, pain characteristics, current treatment, and scapular/thoracic mechanics—were not systematically recorded. Consequently, these variables could not be included in adjusted analyses, and residual confounding cannot be ruled out. Fifth, the sample-size calculation was based on the conventional threshold for a large effect rather than on previous data from tennis players with shoulder pain. Therefore, the study may have been insufficiently powered to detect small or moderate between-group differences. Finally, the findings are specific to tennis players with non-specific shoulder pain and should not be generalized to other athletic populations or to patients with diagnosed structural shoulder disorders.

Future studies should investigate these relationships using longitudinal and interventional designs. Prospective research could determine whether reduced inspiratory muscle strength or altered diaphragm thickening precedes the development of shoulder pain in tennis players. In addition, clinical trials should explore whether adding inspiratory muscle training, breathing retraining, diaphragm-focused manual therapy, or integrated trunk-scapular stabilization exercises to conventional shoulder rehabilitation improves pain, function, and sport performance. Future studies should also include objective measures of scapular kinematics, thoracic mobility, trunk muscle activation, respiratory pattern, and tennis-specific biomechanics to clarify the mechanisms linking diaphragm behavior, kinetic-chain function, and shoulder symptoms.

## 5. Conclusions

The results of this study show that tennis players with non-specific shoulder pain had lower MIP, lower bilateral diaphragm thickness during relaxed inspiration, and lower bilateral diaphragm thickening fraction than asymptomatic players, whereas expiratory diaphragm thickness did not differ between groups. These cross-sectional findings describe group-level differences but do not establish diaphragm dysfunction, causality, or clinical relevance. The findings may also have been influenced by unmeasured demographic, clinical, and sport-specific confounders. Further longitudinal and interventional studies are needed to determine the significance of these findings. Furthermore, these findings apply to the operationally defined study sample and should not be interpreted as representative of any specific clinical shoulder diagnosis.

## Figures and Tables

**Table 1:** Descriptive and clinical data of tennis players with non-specific shoulder pain and asymptomatic tennis players.

Descriptive Data	Tennis Players (*n* = 64)		Mean Difference (95% CI)	*p* Value (Statistic)	Effect Size, Cohen’s d (95% CI)
	Cases (*n* = 32)	Controls (*n* = 32)			
Weight (kg)	72.9 ± 11.0	71.8 ± 13.9	1.13 (−5.14;7.40)	0.719 * (t = 0.361)	0.09 (−0.40; 0.58)
Height (m)	1.77 ± 0.08	1.76 ± 0.09	0.02 (−0.03;0.06)	0.503 * (t = 0.674)	0.17 (−0.32; 0.66)
Age (years)	32.4 ± 12.7	31.8 ± 10.9	0.53 (−5.38; 6.44)	0.957 † (U = 516.50)	0.04 (−0.45; 0.53)
Sex (male/female)	26/6	25/7	n.a.	0.756 ‡ (χ^2^ = 0.097)	0.04 §
QuickDASH (0–100)	21.7 ± 10.1	n.a.	n.a.	n.a.	n.a.
QuickDASH Sports (0–100)	39.8 ± 16.7	n.a.	n.a.	n.a.	n.a.
VAS (0–10)	5.3 ± 1.4	n.a.	n.a.	n.a.	n.a.

Abbreviations: CI, confidence interval; n.a., not applicable. * Student’s *t*-test for independent samples. † Mann–Whitney U test. ‡ Pearson’s chi-square test. § Cramér’s V.

**Table 2:** Outcome measurement differences between tennis players with non-specific shoulder pain and asymptomatic tennis players.

Outcome Measurements	Tennis Players (*n* = 64)		Mean Difference (95% CI)	*p* Value (Statistic)	FDR-Adjusted *p* Value	Effect Size, Cohen’s d (95% CI)
	Cases (*n* = 32)	Controls (*n* = 32)				
MIP (cmH_2_O)	95.65 ± 17.57	111.28 ± 20.05	−15.62 (−25.04;−6.20)	0.002 * (*t* = −3.314)	n.a.	0.82 (0.31; 1.33)
Left Tins thickness (mm)	1.52 ± 0.34	1.83 ± 0.44	−0.31 (−0.50;−0.11)	0.011 † (*U* = 700.500)	0.033	0.78 (0.27; 1.29)
Left Texp thickness (mm)	1.12 ± 0.29	1.26 ± 0.40	−0.14 (−0.32;0.03)	0.356 † (*U* = 580.000)	0.356	0.40 (−0.10; 0.90)
Right Tins thickness (mm)	1.58 ± 0.35	1.87 ± 0.36	−0.29 (−0.47;−0.11)	0.002 * (*t* = −3.220)	0.012	0.81 (0.30; 1.32)
Right Texp thickness (mm)	1.16 ± 0.31	1.29 ± 0.33	−0.12 (−0.29;0.03)	0.073 † (*U* = 644.500)	0.088	0.40 (−0.10; 0.90)
Left DTF (%)	36.06 ± 15.89	47.55 ± 19.61	−11.48 (−20.41; −2.56)	0.018 † (*U* = 335.500)	0.036	0.64 (0.14; 1.14)
Right DTF (%)	36.97 ± 18.32	46.80 ± 16.40	−9.83 (−18.52; −1.14)	0.026 † (*U* = 346.000)	0.039	0.57 (0.07; 1.07)

Abbreviations: CI, confidence interval; DTF, diaphragm thickening fraction; MIP, maximum inspiratory pressure; Texp, thickness at the end of relaxed expiration; Tins, thickness at the end of relaxed inspiration. * Student’s *t*-test for independent samples. † Mann–Whitney U tests. FDR-adjusted *p* values were calculated for the six secondary diaphragm ultrasound outcomes using the Benjamini–Hochberg procedure. MIP was treated as the primary outcome and was not included in the adjustment.

## Data Availability

The raw data supporting the conclusions of this article will be made available by the authors on request.
